# Investigating the causal relationship and mediating mechanisms between Clonal hematopoiesis and vitamin D levels: A Mendelian randomization study

**DOI:** 10.1097/MD.0000000000046608

**Published:** 2025-12-19

**Authors:** Chenliang Liang, Mengqi Li, Zhiwen Sun

**Affiliations:** a Department of Orthopedics, Chifeng Municipal Hospital, Chifeng City, Inner Mongolia, China; b Department of Ultrasound Medicine, Chifeng Municipal Hospital, Chifeng City, Inner Mongolia, China.

**Keywords:** clonal hematopoiesis, inflammatory cytokines, medication, Mendelian randomization, vitamin D levels

## Abstract

Clonal hematopoiesis (CH) is defined by the clonal proliferation of hematopoietic cells that acquire somatic mutations in specific driver genes. Growing evidence suggests that vitamin D (VD), beyond its well-established role in calcium and bone homeostasis, also participates in diverse physiological processes, including cell cycle regulation and immune modulation. Although recent studies have suggested a potential link between CH and VD, the causal nature of this relationship remains to be determined. We conducted a 2-sample Mendelian randomization (MR) study to evaluate the potential causal relationship between CH and VD. Five CH subtypes were analyzed: overall CH (CH-overall), CH with DNMT3A mutations (CH-DNMT3A), CH with TET2 mutations (CH-TET2), large clones (CH-large), and small clones (CH-small). Genetic instruments were derived from genome-wide association studies (GWAS), and the inverse-variance weighted (IVW) method was used as the primary analytical approach. Sensitivity analyses and reverse MR were performed to assess the reliability and directionality of the observed associations. Additionally, a 2-step MR framework was applied to explore potential underlying mechanisms and determine whether inflammatory cytokines mediated the CH–VD relationship. MR analysis identified a statistically significant positive association between CH-large and VD (odds ratio: 1.21, 95% CI: 1.02–1.43, *P* = .026), suggesting a potentially protective effect of large CH clones on VD. No significant associations were found for other CH subtypes. Reverse MR analysis provided no evidence of a bidirectional causal relationship between VD and any CH phenotype. Two-step MR results indicated that MMP-10 may partially mediate the causal link between CH-large and VD, accounting for approximately 8.09% of the total effect. No significant horizontal pleiotropy was detected, supporting the validity of the MR assumptions. Our findings suggest a positive association between large CH clones and VD levels, with MMP-10 potentially serving as a mediator in this pathway. These results provide preliminary evidence for a broader biological interaction between CH and VD. Further research is warranted to elucidate the molecular mechanisms underlying this association and its potential implications in immune and metabolic health.

## 1. Introduction

Clonal hematopoiesis (CH) is defined by the overrepresentation of hematopoietic stem and progenitor cell (HSPC) clones in blood cell formation. The incidence of CH increases markedly with age and can result from somatic mutations in specific genes or from chromosomal abnormalities such as duplications or deletions. These genetic changes that promote clonal expansion may alter the behavior of mature blood cells, contributing to elevated production of pro-inflammatory cytokines.^[1]^ The active metabolite of vitamin D, 1,25-dihydroxyvitamin D3 (1,25(OH)₂D₃), also referred to as calcitriol or VitD3, functions as a central regulator of gene transcription in higher organisms.^[2]^ VitD3 mediates its biological effects through the vitamin D receptor (VDR), a member of the nuclear hormone receptor superfamily.^[3]^ It is involved in multiple physiological processes, including apoptosis, cell proliferation, and immune regulation.^[4]^ As a modulator of both innate and adaptive immunity, VitD3 can regulate systemic inflammation by inhibiting toll-like receptor signaling and reducing the expression of cytokines, adipokines, and chemokines associated with macrophage infiltration into adipose tissue.^[5,6]^

An experimental study using rat bone marrow progenitor cells showed that vitamin D3 enhances the formation of macrophage colonies and clusters under the influence of colony-stimulating factors.^[7]^ In this study, we investigated the potential causal relationship between 5 subtypes of CH phenotypes and vitamin D3 levels using a 2-sample Mendelian randomization (MR) approach. We further evaluated whether inflammatory cytokines mediate this potential relationship. MR leverages genetic variants as instrumental variables (IVs) to infer causality between an exposure and an outcome, thereby reducing biases due to confounding and reverse causation commonly seen in observational studies.^[8]^

## 2. Materials and methods

### 2.1. Study design

**Figure 1.**
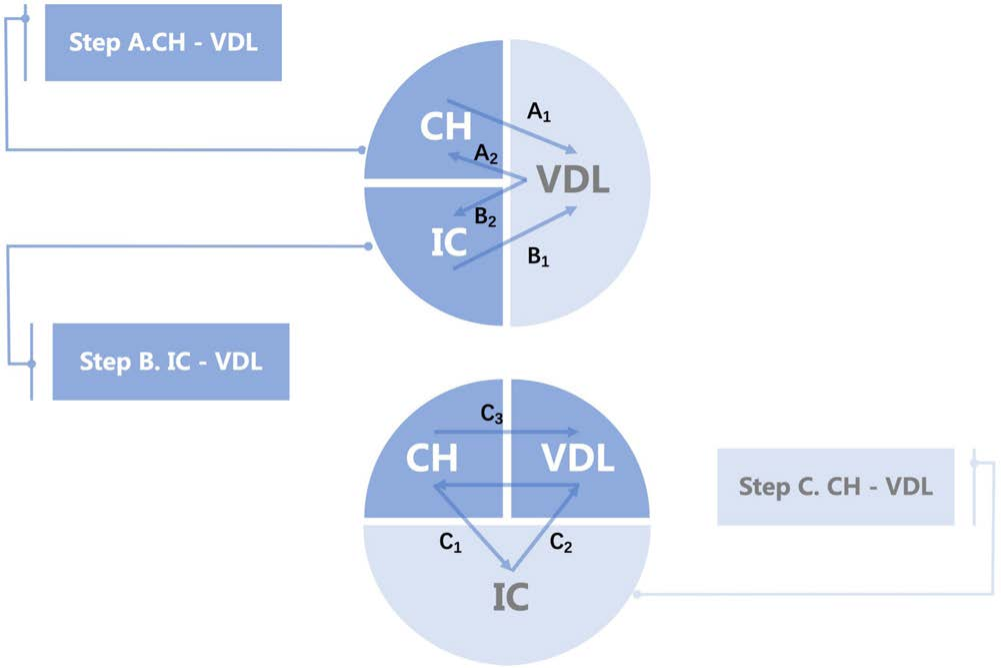
The flow chart of the study. (A) The causal associations between clonal hematopoiesis and vitamin D. (B) The causal associations between inflammatory cytokines and vitamin D. (C) The total effect can be decomposed into: the indirect effect, which is estimated using a 2-step approach (where C1 represents the effect of clonal hematopoiesis on the mediator, and C2 represents the effect of the mediator on vitamin D), as well as through the product method (C1 × C2); and the direct effect, calculated as C3–(C1 × C2). OR = odds ratio.

This study comprises 3 key components, as illustrated in Figure 1: assessing the causal effect of CH on vitamin D levels (VD) (step A_1_); evaluating the causal impact of 91 inflammatory cytokines on VD (step B_1_); and conducting mediation analysis to determine whether inflammatory cytokines mediate the CH–VD relationship (step C). We selected single-nucleotide polymorphisms (SNPs) as IVs. The MR analysis was conducted under the following assumptions: IVs are associated with the exposure (CH phenotypes); IVs are independent of confounding factors; and IVs influence the outcome (VD) only through their effect on the exposure.^[9,10]^

### 2.2. Data sources

Summary statistics for genetic associations with CH were obtained from a large-scale genome-wide association study (GWAS) involving 200,453 individuals of European ancestry from the UK Biobank, as reported by Siddhartha et al.^[11]^ Summary data for overall and subtype-specific CH analyses are publicly accessible via the GWAS Catalog (https://ftp.ebi.ac.uk/pub/databases/gwas/summary_statistics/GCST90102001-GCST90103000/) under the following accession numbers: GCST90102618 (overall CH), GCST90102619 (DNMT3A-CH), GCST90102620 (TET2-CH), GCST90102621 (small clone CH), and GCST90102622 (large-clone CH). The GWAS summary data for VD were sourced from the IEU OpenGWAS project (https://gwas.mrcieu.ac.uk/). Genetic data for 91 inflammatory cytokines were derived from a previously published GWAS involving 14,824 participants.^[12]^ Full per-protein GWAS summary statistics are available at https://www.phpc.cam.ac.uk/ceu/proteins and the EBI GWAS Catalog (accession numbers GCST90274758 to GCST90274848).

All datasets utilized in this research were anonymized, stripped of personal identifiers, and publicly accessible, thus exempting the study from the requirement of ethical review board approval. Furthermore, since the research poses no additional risks to participants, it does not necessitate further informed consent procedures.

### 2.3. Instrumental variables selection

To construct reliable IVs for MR analysis, SNPs significantly associated with each CH phenotype were selected using a threshold of *P* <1 × 10⁻⁵. For VD, we applied a threshold of *P* <5 × 10⁻⁶.^[13]^ To maximize the number of instruments for each inflammatory cytokine, SNPs with *P* <1 × 10⁻⁵ were selected. To ensure independence among SNPs, linkage disequilibrium pruning was performed, retaining only those with *r*² <0.001. This minimized potential correlations and indirect effects among SNPs. Palindromic and ambiguous SNPs were excluded from IVs for MR analysis. The strength of each IV was assessed using the F-statistic, calculated as F = β²/se². An *F*-statistic >10 is generally considered indicative of strong instruments with minimal risk of weak instrument bias.^[14]^

### 2.4. MR analyses

To estimate the causal effects of CH and inflammatory cytokines on VD, we conducted 2-sample MR analyses (step A1 and step B1 in Fig. 1). The inverse-variance weighted (IVW) method was used as the primary analytical approach. Additional analyses were performed using MR-Egger, weighted median, simple mode, and weighted mode methods. MR results were expressed as odds ratios with 95% CIs. Statistical significance was defined as a *P*-value <.05 in the IVW analysis. Reverse MR was also conducted, using VD as the exposure and CH as the outcome, to assess potential bidirectional causality.

### 2.5. Mediation analysis

Based on the results from the 2-sample MR analyses (step A and step B in Fig. 1), CH phenotypes and inflammatory cytokines showing significant causal effects on VD were selected for mediation analysis. We first evaluated whether CH had a causal effect on specific cytokines (step C in Fig. 1). If such an effect was observed, we further assessed whether the cytokine acted as a mediator in the pathway from CH to VD.

### 2.6. Sensitivity analyses

Cochran Q test was used to assess heterogeneity among SNPs. Scatter plots and funnel plots were generated to visualize SNP–exposure and SNP–outcome associations. Leave-one-out analysis was conducted to evaluate whether any single SNP disproportionately influenced the overall MR results.^[15]^ Additionally, MR-Egger regression was applied to detect potential horizontal pleiotropy. All statistical analyses were performed using R software (version 4.4.3) with the TwoSampleMR package (version 0.6.11).

## 3. Results

### 3.1. Instrumental variables selection

As described in the methodology, IVs for CH, 91 inflammatory cytokines, and VD were selected based on GWAS data. In total, 159 SNPs were identified as IVs significantly associated with CH (*F*-statistic range: 19.42–222.55), 223 SNPs for VD (*F*-statistic range: 19.55–1453.73), and 2973 SNPs for inflammatory cytokines (*F*-statistic range: 19.51–1472.93). Detailed characteristics of these IVs are provided in Tables S1–3, Supplemental Digital Content, https://links.lww.com/MD/Q977.

### 3.2. Causal effect of CH on VD

**Figure 2.**
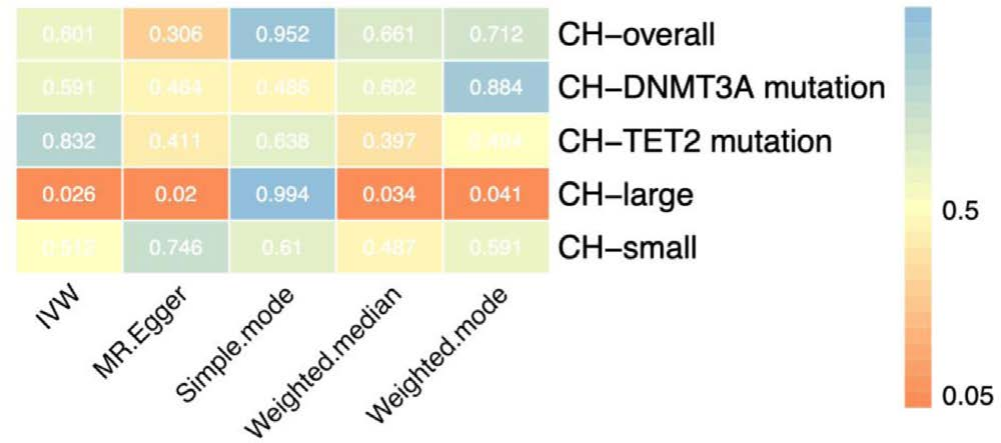
Hot plot of the causal relationship between clonal hematopoiesis and vitamin D. CH-DNMT3A = clonal hematopoiesis with DNMT3A mutations, CH-large = large-clone clonal hematopoiesis, CH-overall = overall clonal hematopoiesis, CH-small = small clone clonal hematopoiesis, CH-TET2 = clonal hematopoiesis with TET2 mutations.

Overall, we found suggestive evidence for a causal association between only 1 CH phenotype and VD (Fig. 2 and Fig. S1, Supplemental Digital Content, https://links.lww.com/MD/Q976). Specifically, large-clone CH showed a positive association with VD levels based on IVW analysis (OR = 1.210, 95% CI = 1.023–1.431; *P* = .026). The MR-Egger and weighted median methods supported this result. The intercept from the MR-Egger regression indicated no evidence of horizontal pleiotropy. Detailed results are presented in Fig. S2, Supplemental Digital Content, https://links.lww.com/MD/Q976. No statistically significant reverse causal associations between VD and CH were observed using MR-Egger, weighted median, IVW, simple mode, or weighted mode methods (*P* >.05) (Additional file: Table S4, Supplemental Digital Content, https://links.lww.com/MD/Q977 in the supplementary materials).

### 3.3. Causal effect of inflammatory cytokines on VD

**Figure 3.**
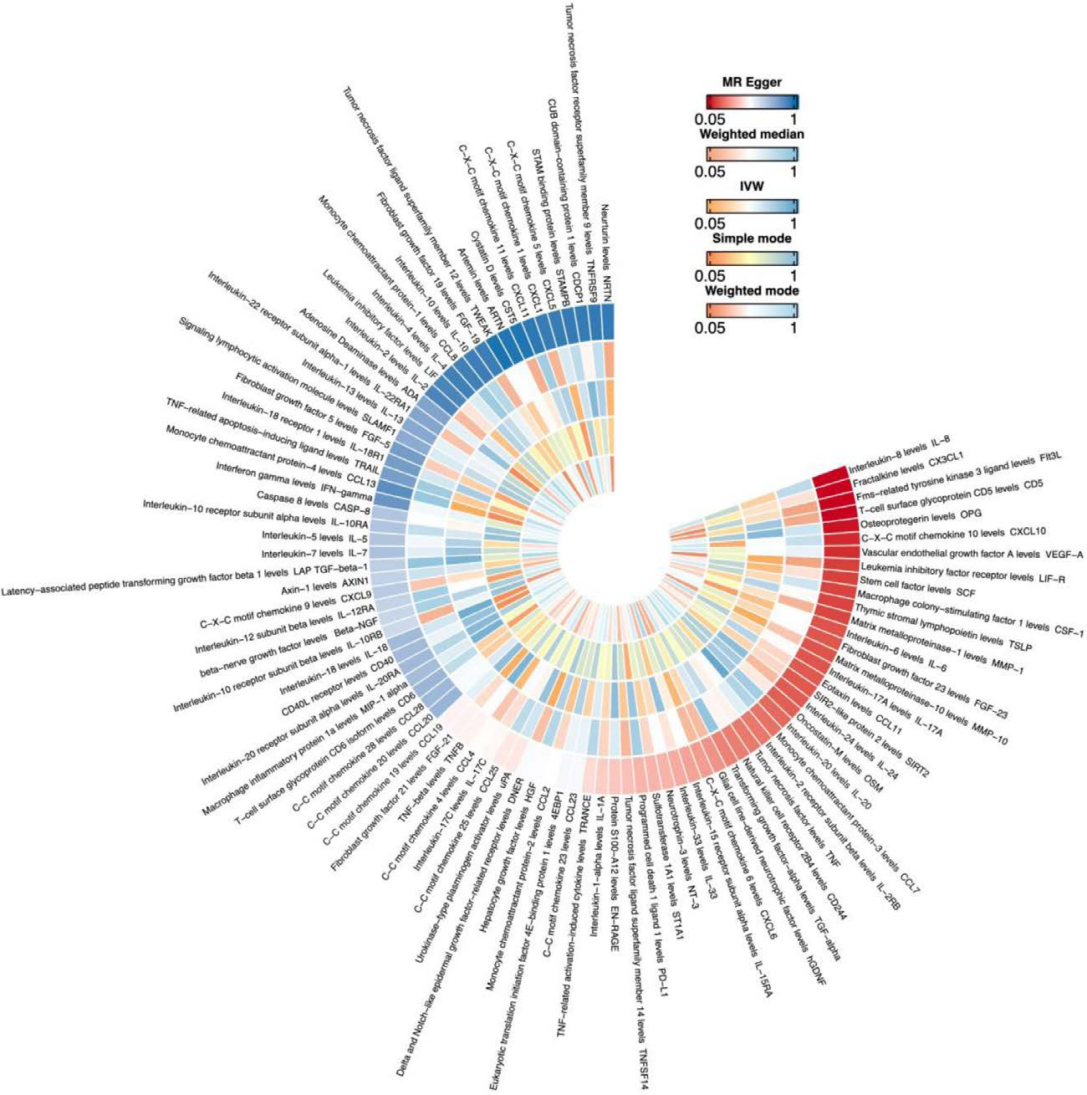
Ringhot plot of the causal relationship between inflammatory cytokines and vitamin D.

In summary, 4 inflammatory proteins showed potential associations with VD levels (Figs. 3 and Fig. S3, Supplemental Digital Content, https://links.lww.com/MD/Q976). Specifically, elevated levels of leukemia inhibitory factor receptor (LIF-R) (OR = 1.027, 95% CI = 1.009–1.045; *P* = .003) and monocyte chemoattractant protein-4 (CCL13) (OR = 1.013, 95% CI = 1.001–1.025; *P* = .034) were associated with increased VD levels. Conversely, higher levels of matrix metalloproteinase-10 (MMP-10) (OR = 0.990, 95% CI = 0.981–0.999; *P* = .027) and delta and notch-like epidermal growth factor-related receptor (DNER) (OR = 0.981, 95% CI = 0.965–0.998; *P* = .028) were linked to decreased VD levels. No evidence of horizontal pleiotropy was detected for these 4 cytokines (*P* >.05). Detailed findings are provided in Fig. S4, Supplemental Digital Content, https://links.lww.com/MD/Q976. No significant associations were observed in the reverse MR analysis between VD and these cytokines (Additional file: Table S5, Supplemental Digital Content, https://links.lww.com/MD/Q977 in the supplementary materials).

### 3.4. Mediation effect of circulating inflammatory cytokines

**Figure 4.**
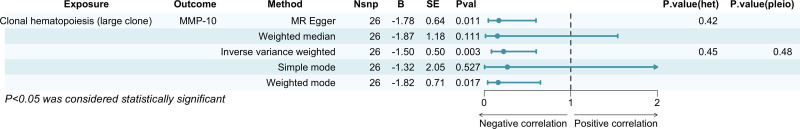
Forest plot of the causal relationship between clonal hematopoiesis and inflammatory cytokines. An OR >1 suggests a positive association between the exposure and the outcome, whereas an OR <1 indicates a negative association. MMP-10 = matrix metalloproteinase-10, OR = odds ratio.

**Figure 5.**
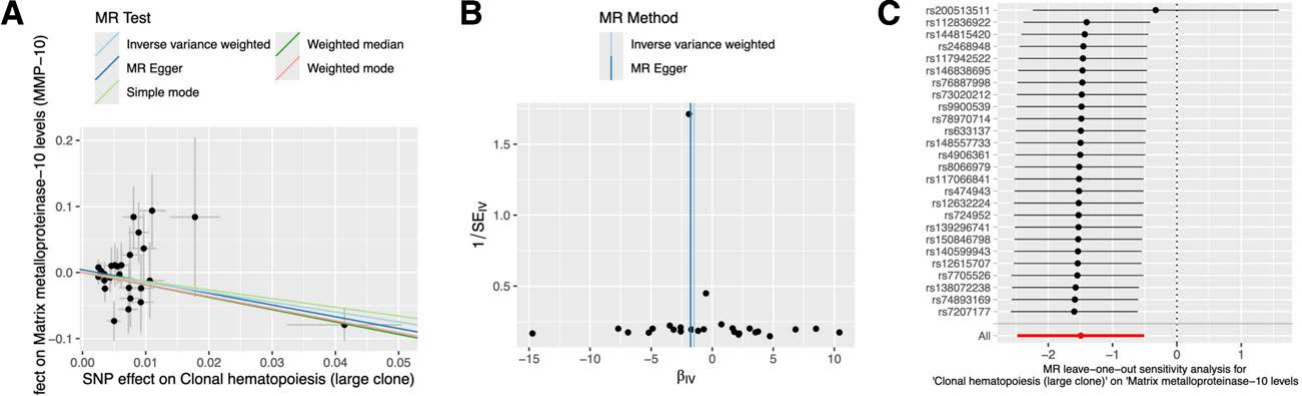
Sensitivity analyses of the association between clonal hematopoiesis and inflammatory cytokines. Scatter plot, funnel plot, and leave-one-out analysis depicting the causal associations between inflammatory cytokines and vitamin D levels. (A) scatter plot; (B) funnel plot; (C) leave-one-out plot.

We conducted a 2-step MR analysis to identify potential mediators and quantify the indirect effects of CH on vitamin D (VD) levels. As illustrated in Figure 1 (Step C), both large-clone CH and MMP-10 demonstrated significant causal associations with VD levels. Additionally, a negative correlation was observed between large-clone CH and MMP-10 (*P* = .003). The detailed results are summarized in Figure 4. Notably, MMP-10 was found to mediate approximately 8.09% of the total effect of CH on VD, as presented in Table 1. No evidence of significant heterogeneity or horizontal pleiotropy was detected in the relationship between large-clone CH and MMP-10, with further details provided in Figure 5.

**Table 1:** Mediation analysis of the mediation effect of CH on vitamin D level.

Exposure	Outcome	Mediator	Total effect	Direct effect	Mediation effect
			Effect size (Beta)		Mediated (%)
CH (large-clone)	Vitamin D levels	MMP-10	0.191	0.175	17.53

CH = clonal hematopoiesis.

## 4. Discussion

To the best of our knowledge, this study marks the first utilization of MR to investigate the causal link between CH and vitamin D (VD), while also assessing inflammatory cytokines as potential mediators. Our thorough MR analysis demonstrated that only 1 CH subtype has a meaningful impact on vitamin D concentrations. Among the 91 inflammation-related cytokines examined, 4 – delta and notch-like epidermal growth factor-related receptor (DNER), leukemia inhibitory factor receptor (LIF-R), monocyte chemoattractant protein-4 (CCL13), and matrix metalloproteinase-10 (MMP-10) – showed a statistically significant association with vitamin D levels. Importantly, MMP-10 was the sole cytokine identified to mediate the causal relationship between CH and VD.

CH is defined by the clonal expansion of hematopoietic stem cells and their offspring, driven by somatic mutations that confer a selective growth advantage. This condition is highly prevalent, affecting more than 1-third of the general population.^[16,17]^ Within the hematopoietic system, specific genetic mutations enhance the survival and proliferation of hematopoietic stem cells and their progeny, ultimately leading to CH – a condition strongly associated with aging and commonly seen in older individuals.^[18,19]^ In addition to its well-established roles in calcium homeostasis and bone metabolism, vitamin D3 has also been implicated in immune modulation.^[20]^ The active metabolite of vitamin D3, 1,25-dihydroxyvitamin D3 [1,25(OH)_₂_D_₃_], mediates its biological actions through the vitamin D receptor (VDR), playing a key role in immune regulation.^[21]^ The immunomodulatory effects of vitamin D3 derivatives are well supported by existing scientific evidence.

A study by Elias et al showed that 1,25(OH)_₂_D_₃_ facilitates the clonal formation of macrophage colonies and aggregates derived from rat bone marrow progenitor cells in the presence of colony-stimulating factors.^[7]^ Although CH is primarily defined by the presence of specific somatic mutations, its progression is also shaped by environmental influences^[22–24]^ and inherited genetic predispositions,^[25,26]^ the underlying mechanisms of which remain poorly understood. Moreover, due to the complex biological nature of CH, conventional observational studies have struggled to clarify its potential impact on vitamin D3 levels.

MR is a statistical method used to evaluate causal relationships between an exposure and an outcome, particularly when randomized controlled trials are not feasible or when observational data may be affected by confounding or reverse causation.^[10]^ In this study, MR analysis was employed to investigate the potential causal associations between CH and circulating vitamin D levels.

The evidence regarding whether specific CH subtypes influence vitamin D levels (VDL) remains inconclusive. MR results suggest a positive association between large-clone CH and VDL. However, few studies have specifically explored the causal relationship between large-clone CH and VDL. To further investigate the underlying mechanisms, a reverse MR analysis was conducted, which did not identify statistically significant causal effects between large-clone CH and VDL.

Recent studies have highlighted the role of CH-related mutations in immune regulation, particularly within the innate immune system. For example, Tet2-deficient murine macrophages produce elevated levels of inflammatory cytokines such as IL-1β and IL-6 when exposed to low-density lipoprotein or bacterial endotoxin, compared to wild-type macrophages.^[27–29]^ Similarly, individuals with TET2 mutations have been found to have increased circulating levels of IL-6 and IL-1β.^[25,30]^ Emerging evidence also indicates that DNMT3A mutations can affect innate immune responses. For instance, mast cells from Dnmt3a-deficient mice exhibit heightened activity during allergic responses, characterized by increased production of IL-6, tumor necrosis factor α, and IL-13 following immunoglobulin E stimulation.^[31]^

Notably, CH-associated mutations are predominantly observed in circulating granulocytes, monocytes, and natural killer cells, but are rarely present in B cells and infrequently detected in T cells.^[32]^ DNMT3A mutations have been reported in the T cells of 30% to 50% of individuals with DNMT3A-mutated clonal hematopoiesis of indeterminate potential.^[32,33]^ One possible explanation is that these mutations may alter hematopoietic stem cell differentiation away from the T-cell lineage or occur in progenitor cells that have already lost the potential to differentiate into T cells. Alternatively, T lymphopoiesis may have significantly declined by middle age, which is typically when CH clones arise. Despite the relatively low frequency of CH mutations in lymphoid cells, accumulating evidence suggests that these genes play essential roles in T- and B-cell function. For example, mutations in TET2 and DNMT3A are frequently observed in CD4 + T-helper cell-derived lymphomas, such as angioimmunoblastic T-cell lymphoma.^[34]^

There is growing evidence that 1,25(OH)_₂_D_₃_ plays a key role in immune regulation. For instance, it demonstrates potent antiproliferative effects on T cells following mitogen activation, partly through the upregulation of inhibitory receptors such as CTLA-4.^[35,36]^ Additionally, it inhibits the proliferation of both lymphoid and myeloid leukemia cell lines by modulating cell cycle regulators, including upregulation of p21 and p27 and downregulation of CDK2/4, cyclin D1, and cyclin A.^[37]^ Beyond its antiproliferative effects, 1,25(OH)_₂_D_₃_ also promotes apoptosis by suppressing BCL2 expression, thereby increasing lymphocyte susceptibility to programmed cell death.^[38,39]^ Vitamin D has also been shown to influence T-cell differentiation toward a less inflammatory profile. Specifically, 1,25(OH)_₂_D_₃_ reduces IFN-γ production while increasing IL-4 secretion.^[40]^ Furthermore, it enhances both the development and immunosuppressive activity of Foxp3 + CD4 + regulatory T cells.^[41]^ Recent studies further indicate that 1,25(OH)_₂_D_₃_ suppresses IL-17 production by T cells.^[10,11]^ Consistent with these findings, other studies have shown that 1,25(OH)_₂_D_₃_ inhibits Th17 cell differentiation.^[6]^ Physiologically relevant concentrations of 1,25(OH)_₂_D_₃_ also suppress the production of IL-17, IL-21, and IL-22 in Th17-polarized T cells, suggesting that these effects are mediated through the vitamin D receptor (VDR) transcription factor complex.

1,25(OH)_₂_D_₃_ interacts with the immune system through its regulatory effects on the differentiation and function of immune cells such as lymphocytes, macrophages, and natural killer cells, as well as by modulating cytokine production in both in vivo and in vitro settings. Notable immunomodulatory and immunosuppressive effects of 1,25(OH)_₂_D_₃_, observed in experimental models, include:

1) Reduced production of pro-inflammatory cytokines such as IL-2, IL-6, and IL-12;
2) Decreased interferon-γ (IFN-γ) secretion;
3) Suppressed tumor necrosis factor expression;
4) Increased production of antiinflammatory cytokines, including IL-4, IL-5, and IL-10;
5) Inhibition of autoantibody production by B lymphocytes.^[42,43]^

There is strong evidence indicating that both CH and vitamin D (VD) are involved in the regulation of the immune system, suggesting a potential functional relationship between the two. To investigate whether inflammatory cytokines mediate this relationship, we applied a 2-sample MR design. Our results show that CH-associated with TET2 mutations is significantly linked to elevated levels of monocyte chemoattractant protein-4 (CCL13) (*P* = .044), while large-clone CH exhibits a negative association with matrix metalloproteinase-10 (MMP-10) concentrations (*P* = .03). Furthermore, a 2-step MR analysis was conducted to assess the mediating role of MMP-10 in the pathway from large-clone CH to VD. Notably, MMP-10 was found to mediate approximately 17.54% of the total effect.

The precise mechanism by which MMP-10 influences the causal relationship between large-clone CH and VD remains to be fully elucidated. MMP-10, also referred to as stromelysin-2, plays a role in physiological processes such as bone development.^[44]^ Although MMP-10 is typically undetectable in most healthy tissues, it is rapidly induced in response to tissue injury or inflammatory stimuli.^[45–47]^

Matrix metalloproteinases (MMPs) constitute a family of zinc- and calcium-dependent endopeptidases that are crucial for extracellular matrix remodeling. Among them, collagenases such as MMP-1, MMP-8, MMP-13, and MMP-14, as well as gelatinases MMP-2 and MMP-9, are primarily responsible for degrading fibrillar collagen. MMP-3 acts as a key activator of other MMPs, including MMP-1 and MMP-9.^[48–50]^ Under normal physiological conditions, MMPs are not expressed in noncalcified, healthy tissues; however, they are upregulated in activated cells, where they contribute to tissue remodeling and repair. Moreover, MMPs modulate the innate immune response by influencing cytokine and chemokine processing, apoptosis, and antimicrobial peptide activation.^[51]^

Evidence regarding the association between vitamin D and MMPs in human populations remains limited. Wasse et al reported an inverse correlation between serum MMP-9 and 25-hydroxyvitamin D_₃_ [25(OH)D_₃_] levels, but no significant association was observed between MMP-2 and 25(OH)D_₃_. Daria et al found that circulating MMP-10 levels are inversely correlated with serum 25(OH)D_₃_ concentrations in patients with type 2 diabetes (T2D), particularly those with vitamin D deficiency or chronic kidney disease.^[52]^ Observational studies suggest a possible link between MMP-10 and VD. However, the causal direction of this relationship remains uncertain. MR analysis revealed a negative association between MMP-10 and VD, with no evidence of a significant reverse causal effect.

Vitamin D insufficiency is widely prevalent across all age groups worldwide,^[53,54]^ establishing it as a critical public health challenge. This deficiency plays a central role in the pathogenesis of numerous chronic endocrine and metabolic disorders. Meta-analytical evidence consistently demonstrates that adequate vitamin D levels in adults are associated with a reduced risk of cardiovascular diseases, type 2 diabetes, and metabolic syndrome.^[55]^ In recent years, research has increasingly emphasized the long-term consequences of suboptimal vitamin D status during key developmental periods – particularly childhood and adolescence – including impaired skeletal maturation, elevated risks of osteoporosis and osteoarthritis later in life, and increased susceptibility to metabolic, cardiovascular, respiratory, and psychiatric conditions.^[56]^

CH was initially regarded primarily in the context of hematologic malignancies; however, advances in genetic sequencing technologies have revealed that clonal expansions can also arise in individuals without overt disease.^[17]^ Although these mutations are often indicative of benign clonal dynamics, their full clinical implications remain incompletely understood.^[57]^ CH has been linked not only to hematologic disorders but also to a broad spectrum of nonhematologic conditions, particularly those mediated by chronic inflammation.^[58]^ Furthermore, CH has been implicated in type 2 diabetes, obesity, multiple solid tumors, peripheral artery disease, and severe manifestations of COVID-19, among other comorbidities.^[59–61]^

As outlined, the causal relationship between CH and vitamin D (VD) is a complex phenomenon involving multiple interacting mechanisms and mediators. Future preclinical studies should focus on identifying the molecular drivers underlying both CH and VD, as well as on refining and developing more physiologically relevant animal models. Given that genetic variants are randomly assigned at conception, MR studies are less prone to confounding and reverse causation – key limitations of conventional observational studies.^[62]^ Furthermore, MR methods are not subject to subjective biases such as self-reporting or recall errors, thereby substantially reducing the risk of information bias.^[63]^

Although the study was conducted with methodological rigor, several limitations should be acknowledged. First, the genetic data were obtained exclusively from individuals of European ancestry, which may limit the generalizability of the findings to populations of other ethnic and geographic backgrounds. Second, the conclusions are based entirely on observational genome-wide association studies (GWAS) and have not been confirmed through experimental or clinical validation; therefore, additional in vivo and clinical investigations are necessary to substantiate these associations. Third, while we analyzed 5 subtypes of CH, other subtypes not included in this study may also be involved in the regulation of vitamin D. Furthermore, our analysis focused on 91 inflammatory cytokines, yet other cytokines not examined in this research might also influence the relationship between gut microbiota and vitamin D levels. Finally, although 3 inflammatory cytokines were identified as mediators in the causal pathway from CH to vitamin D (VD), the underlying biological mechanisms remain unclear and require further mechanistic studies to understand their roles in vitamin D metabolism fully.

## 5. Conclusion

In summary, MR findings support a potential link between large-clone CH and VD. However, the underlying biological mechanisms remain incompletely understood. Mediation analysis within the MR framework suggests that MMP-10 may act as an intermediary in the pathway connecting large-clone CH and VD. Leveraging the strengths of MR methodology, our findings contribute to a deeper understanding of the biological interplay between large-clone CH and VD. Clarifying the causal relationship between these factors and identifying potential mediators may inform novel therapeutic strategies and enhance our understanding of their roles in metabolic and immune regulatory networks.

## Acknowledgments

We gratefully acknowledge the contributions of all GWAS authors and participants, whose publicly available summary statistics were essential for the successful completion of this research.

## Author contributions

**Conceptualization:** Chenliang Liang, Mengqi Li.

**Data curation:** Chenliang Liang.

**Formal analysis:** Chenliang Liang.

**Investigation:** Chenliang Liang, Mengqi Li.

**Methodology:** Chenliang Liang, Mengqi Li.

**Project administration:** Mengqi Li, Zhiwen Sun.

**Software:** Chenliang Liang.

**Supervision:** Zhiwen Sun.

**Validation:** Chenliang Liang.

**Visualization:** Chenliang Liang.

**Writing – original draft:** Chenliang Liang, Mengqi Li.

**Writing – review & editing:** Chenliang Liang, Mengqi Li, Zhiwen Sun.

## Supplementary Material




