# Supplementary material for: Investigating the causal relationship and mediating mechanisms between Clonal hematopoiesis and vitamin D levels: A Mendelian randomization study
Source: Medicine (Baltimore). 2025 Dec 19;104(51):e46608. doi: 10.1097/MD.0000000000046608 (PMC12727322; doi:10.1097/MD.0000000000046608)

**S1 Fig. Forest plot of the causal relationship between clonal hematopoiesis and vitamin D.**


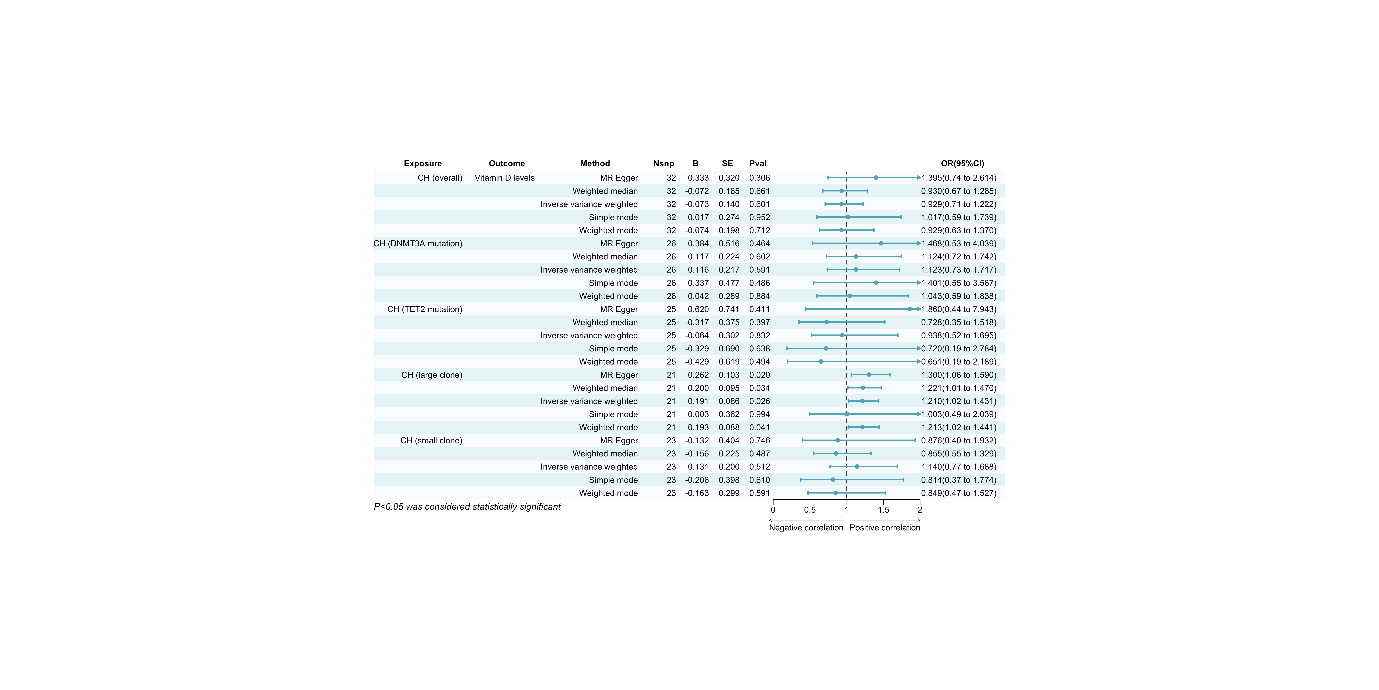


**S2 Fig.** **Sensitivity analyses of the causal relationships between clonal hematopoiesis and vitamin D.** (A) Scatter plot; (B) Funnel plot; (C) Leave-one-out plot.


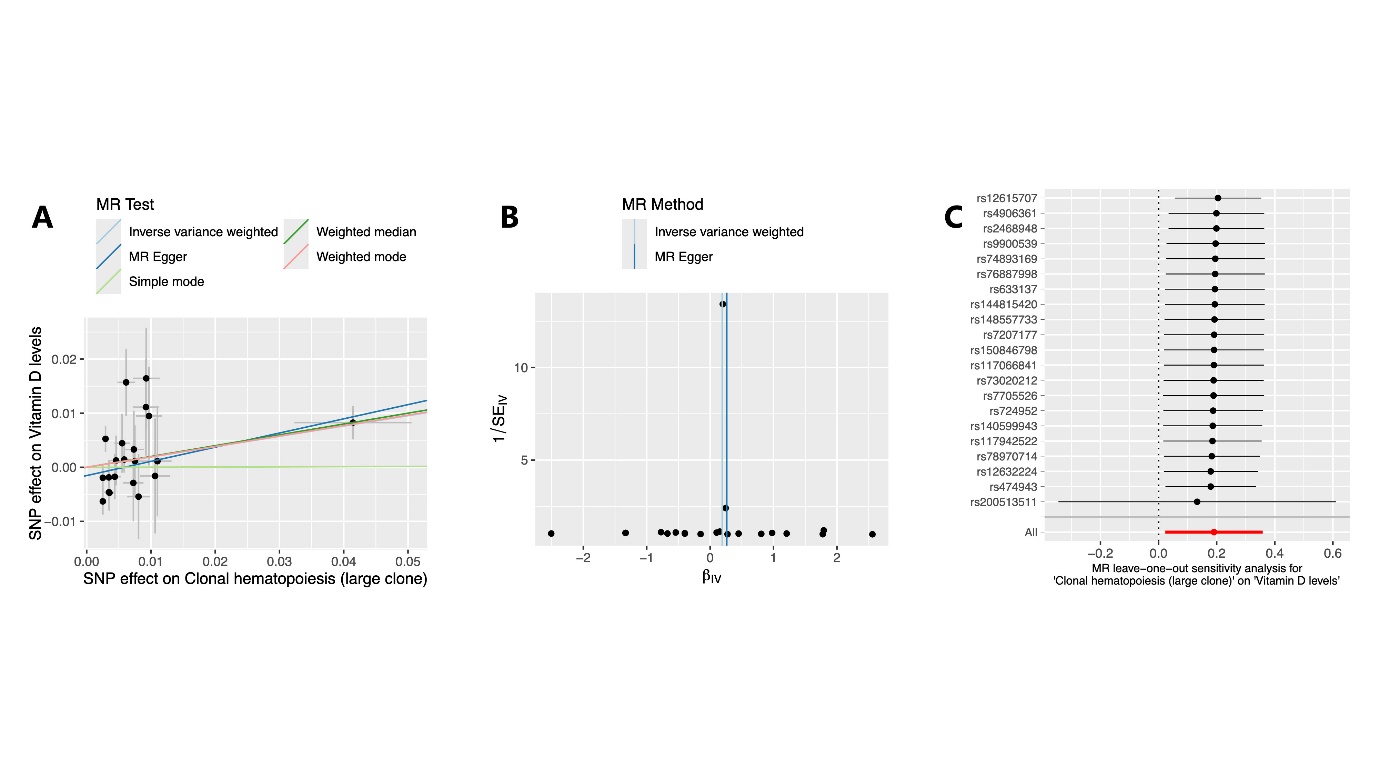


**S3 Fig. Forest plot of the causal relationship between inflammatory cytokines and vitamin D.**


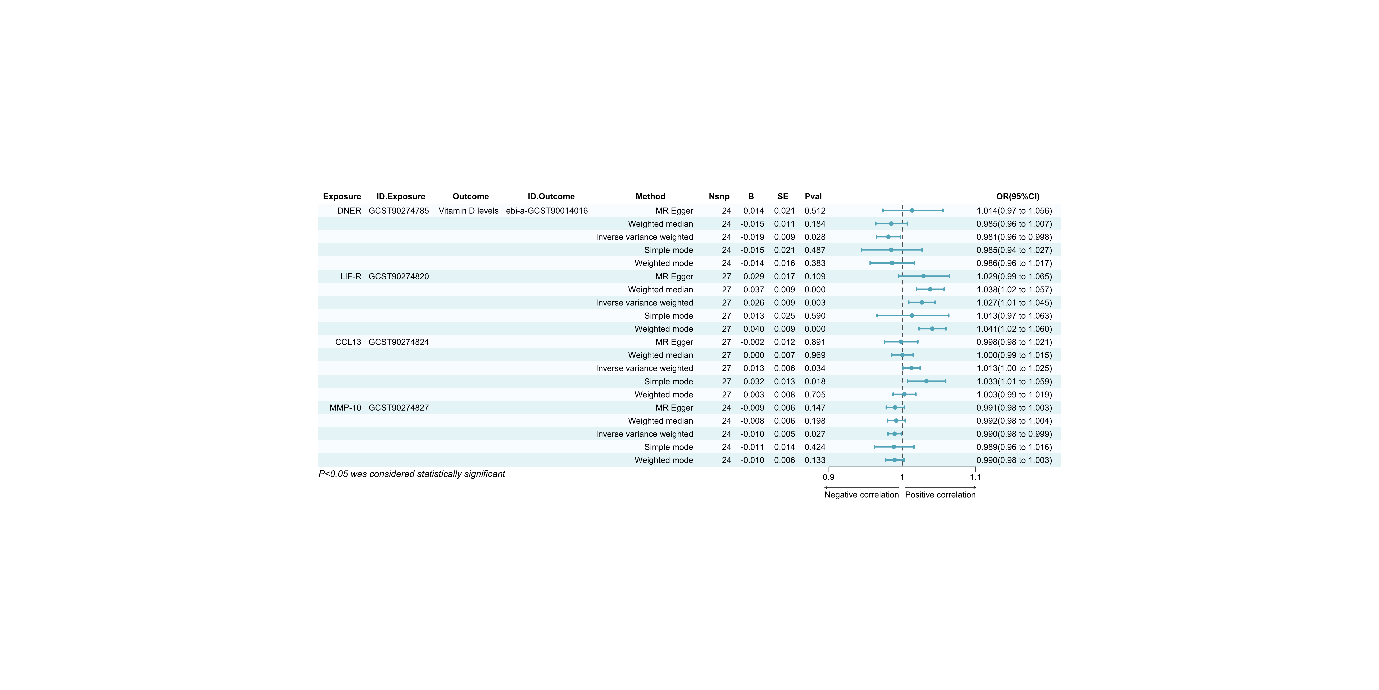


**S4 Fig. Sensitivity analyses of the causal relationships between inflammatory cytokines and vitamin D.** (A) Scatter plot; (B) Funnel plot; (C) Leave-one-out plot.


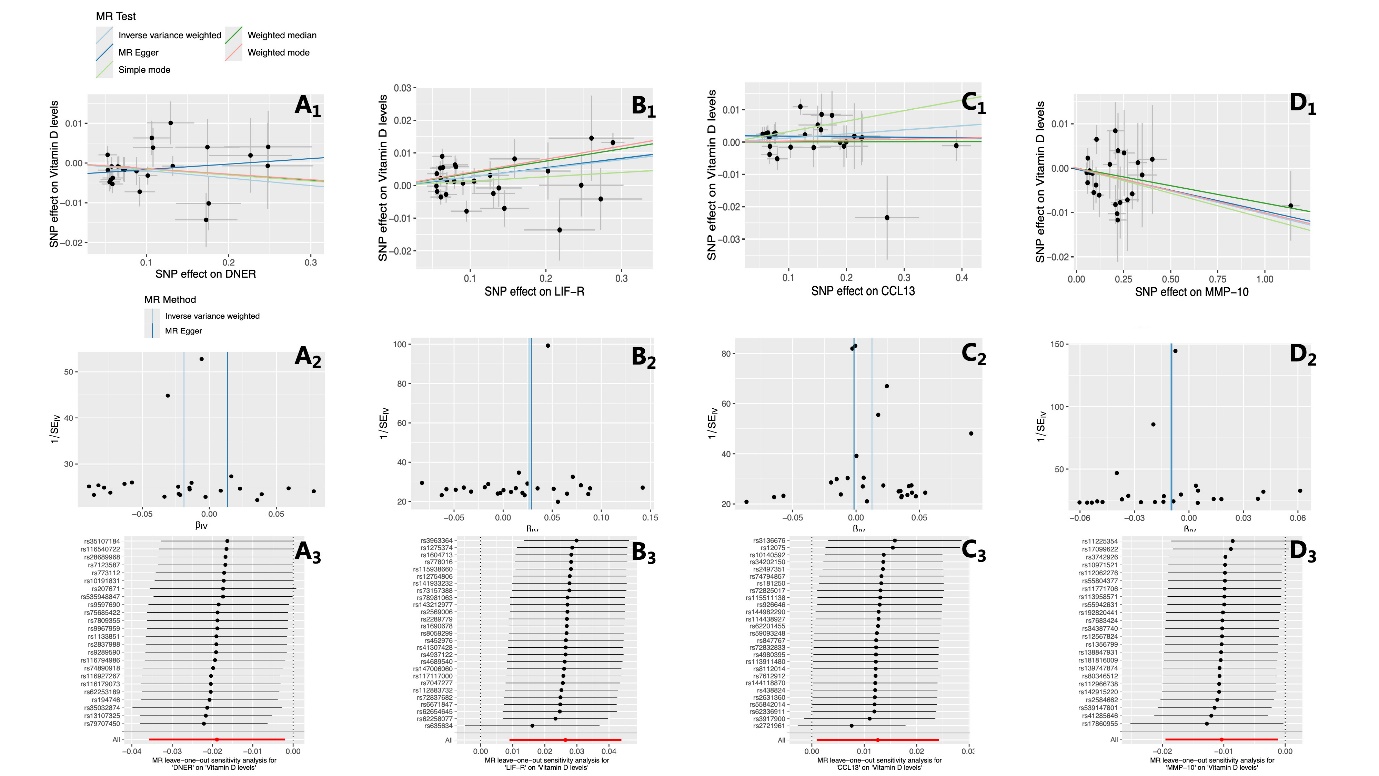

Supplement: Supplementary file 2 [file medi-104-e46608-s002.docx]
